# Identification of host proteins associated with HIV-1 preintegration complexes isolated from infected CD4^+ ^cells

**DOI:** 10.1186/1742-4690-7-66

**Published:** 2010-08-11

**Authors:** Nidhanapati K Raghavendra, Nikolozi Shkriabai, Robert LJ Graham, Sonja Hess, Mamuka Kvaratskhelia, Li Wu

**Affiliations:** 1 Center for Retrovirus Research, Department of Veterinary Biosciences, The Ohio State University, Columbus, Ohio 43210, USA; 2 Center for Retrovirus Research and Comprehensive Cancer Center, College of Pharmacy, The Ohio State University, Columbus, Ohio 43210, USA; 3 Proteome Exploration Laboratory, Beckman Institute, California Institute of Technology, Pasadena, California 91125, USA

## Abstract

An integrated HIV-1 genomic DNA leads to an infected cell becoming either an active or a latent virus-producing cell. Upon appropriate activation, a latently infected cell can result in production of progeny viruses that spread the infection to uninfected cells. The host proteins influence several steps of HIV-1 infection including formation of the preintegration complex (PIC), a key nucleoprotein intermediate essential for integration of reverse transcribed viral DNA into the chromosome. Much effort has gone into the identification of host proteins contributing to the assembly of functional PICs. Experimental approaches included the use of yeast two-hybrid system, co-immunoprecipitation, affinity tagged HIV-1 viral proteins and *in vitro *reconstitution of salt-stripped PIC activity. Several host proteins identified using these approaches have been shown to affect HIV-1 replication in cells and influence catalytic activities of recombinant IN *in vitro*. However, the comprehensive identification and characterization of host proteins associated with HIV-1 PICs of infected cells have been hindered in part by the technical limitation in acquiring sufficient amount of catalytically active PICs. To efficiently identify additional host factors associated with PICs in infected cells, we have developed the following novel approach. The catalytically active PICs from HIV-1-infected CD4^+ ^cells were isolated using biotinylated target DNA, and the proteins selectively co-purifying with PICs have been analyzed by mass spectrometry. This technology enabled us to reveal at least 19 host proteins that are associated with HIV-1 PICs, of which 18 proteins have not been described previously with respect to HIV-1 integration. Physiological functions of the identified proteins range from chromatin organization to protein transport. A detailed characterization of these host proteins could provide new insights into the mechanism of HIV-1 integration and uncover new antiviral targets to block HIV-1 integration.

## Findings

Human immunodeficiency virus type 1 (HIV-1) integrase (IN) is a 288 amino-acid protein with three functional domains: N-terminal domain (NTD), catalytic core domain (CCD) and C-terminal domain (CTD). The NTD contains a zinc binding motif, the CCD has three acidic residues, D64, D116 and E152, which co-ordinate the catalytic divalent metal ions; and the CTD is suggested to nonspecifically bind the DNA substrate [1]. IN catalyzes two endonucleolytic reactions - 3' processing: the removal of two deoxynucleotides from viral DNA ends; and DNA strand transfer: the covalent ligation of viral DNA 3' ends to host chromosomal DNA. While a recombinant IN can catalyze 3' processing and strand transfer reactions [2], the activity of HIV-1 integrase in the context of preintegration complex (PIC) is assisted and modulated by several host factors during proviral DNA formation. The PIC is thought to be derived from the reverse transcription complex and consists of the full length viral DNA and both viral and host proteins that participate in generation of the proviral DNA [3,4].

The PIC formed following reverse transcription is in limiting amounts to permit biochemical purification of the pure complexes and identification of constituent proteins [5]. Previous studies to identify IN-interacting host proteins have primarily used yeast two-hybrid system and co-immunoprecipitations involving ectopically expressed viral and host proteins (Table 1). Another approach has been the *in vitro *reconstitution of salt-stripped PIC activity (PICs treated with high salt result in integration-defective complexes) using purified or recombinant host proteins. These approaches have helped to identify host proteins that physically interact with HIV-1 IN or stimulate HIV-1 IN catalytic activity. A recent study involving use of a biotinylated IN as a tool to detect interacting host proteins concluded that activity of the modified IN was adversely affected [6].

**Table 1:** Summary of previously characterized host proteins interacting with HIV-1 IN

Host proteins	Methods	References
BAF	SS	[11]

Gemin2	IP	[14]

HAT p300	IP	[26]

HMGA1	SS	[8]

HSP 60	PD	[27]

Human EED protein	THS; PD	[28]

Importin 7	IP	[13]

Integrase interactor 1	THS	[29]

LEDGF/p75	IP	[30]

UNG2	PD	[31]

A list of host proteins interacting with HIV-1 integrase and the experimental procedure used to identify the protein-protein interaction. THS: two hybrid system; PD: Pull down; SS: reconstitution of salt stripped PIC activity; IP: immunoprecipitation.

**Figure 1:**
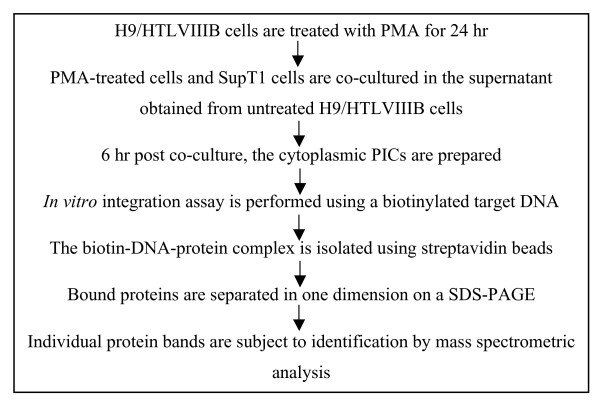
**Experimental design for the identification of host proteins associated with HIV-1 PIC**. The PICs were generated following a protocol described previously [5]. PICs covalently bound to biotinylated DNA were isolated using streptavidin beads. The proteins in the isolated complex were identified by mass spectrometric analysis. PMA: phorbol 12-myristate 13-acetate.

**Figure 2:**
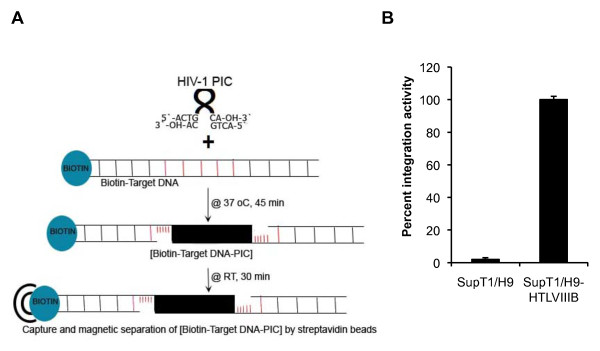
**Isolation of HIV-1 PICs and their activity**. (A) Magnetic separation of functional HIV-1 PICs. The PIC integrates HIV-1 DNA into the ~1.5 kb biotinylated non-viral DNA from pNL4-3 plasmid that serves as a target DNA in the *in vitro *assay. The biotin-target DNA-protein complex is then isolated using streptavidin magnetic beads after incubation at room temperature (RT) for 30 minutes. (B) Integration activity of HIV-1 PICs. The integration activity of the PICs isolated from SupT1/H9-HTLVIIIB cell co-cultures (HIV-1-infected, set to 100 percent) and control cytoplasmic extract from SupT1/H9 cell co-cultures (control cells), using biotin-target DNA, is shown. The analysis was performed as described previously [5].

In the current study, a novel approach to identify the host proteins associated with PIC is presented. The protocol involves using a biotinylated target DNA in the standard *in vitro *PIC reaction assay, and the isolation of the protein complex covalently attached to target DNA using streptavidin beads (Figure 1). As a stable complex that is imported into the nucleus for integration into host chromosome, it is possible that the proteins associated with the HIV-1 DNA remain bound even after catalysis of the integration into a biotinylated target DNA. This assumption is the basis of the approach described here. A well-established protocol has been used to isolate the cytoplasmic PICs (which is a cytoplasmic extract of HIV-1-infected cells) and perform an *in vitro *integration assay [6]. The H9/HTLVIIIB cell line is a chronically HIV-1 infected H9-derived CD4^+ ^cell line that releases infectious HIV-1 into the culture supernatant [7]. Stimulation of the H9/HTLVIIIB cell line with phorbol 12-myristate 13-acetate (PMA) increases viral production several fold and also increases the cell-to-cell transmission of the virus in co-culture experiments [6]. The parental H9 cells that do not produce HIV-1 were used as a negative control. Co-culture of HIV-1 producing H9/HTLVIIIB cells with HIV-1 susceptible cells such as CD4^+ ^SupT1 cells typically leads to a high proportion of infected cells. SupT1 cells (2.5 × 10^9^) were co-cultured for 6 hours with PMA-treated H9/HTLVIIIB or H9 cells (2.5 × 10^8^) in the supernatant (600 ml) obtained from a 24 hour culture of H9/HTLVIIIB or H9 cells, respectively. (All cells were grown to a density of 1-1.5 × 10^6 ^per ml prior to co-culture). The PICs isolated from such co-cultured cells were demonstrated to exhibit high integration activity into naked plasmid DNA [8]. The cytoplasmic PICs generated here (isolated in 50 ml of digitonin-containing lysis buffer) have been used for *in vitro *integration into a ~1.5 kb biotinylated target DNA (100 μg, prepared by PCR amplification of non-viral DNA in pNL4-3 plasmid using the following primer pair: Biotin - 5' CAA AGT GCT GGG ACA ACC GGG 3' and 5' GCG CTC GGC CCT TCC GGC TGG C 3'). At the end of the integration assay, the biotinylated target DNA-PIC complex was bound to streptavidin magnetic beads (MyOne Streptavidin T1 beads, Invitrogen) at room temperature for 30 minutes (Figure 2A). To efficiently remove the majority of non-specific proteins bound to the streptavidin beads, 10 washes of 15 ml each were performed using the assay buffer [5]. The use of a biotinylated target DNA to isolate the PIC precludes the requirement of a tagged HIV-1 protein, and the potential alteration of protein-protein interactions or activity caused by a tagged-protein. The isolation of the DNA-protein complex based on the catalytic activity of PIC makes it possible to identify physiologically relevant viral-host protein interactions.

The integration activity of the isolated complex was confirmed by real-time PCR analysis using primers specific to the target and viral DNA [5]. As expected, no activity was detected either in the absence of a target DNA or with the cytoplasmic extract from the SupT1-H9 co-culture as compared to the activity of PICs isolated from the SupT1-H9/HTLVIIIB co-culture (set to 100%) (Figure 2B). Proteins from complex mixtures such as cell lysates have been identified successfully by using mass spectrometric (MS) techniques [9]. The proteins from the complexes bound to streptavidin beads were eluted by boiling the beads in 30 μl of the SDS-PAGE running buffer at 95°C for 5 minutes. The eluted-boiled proteins were loaded into a single well of a 4-15% gradient SDS-PAGE gel (Bio-Rad), and the proteins were separated in one dimension. Differences between the SupT1-H9/HTLVIIIB and SupT1-H9 co-culture samples could not be readily delineated from visual inspection of Coomassie Blue stained gels. This is not surprising considering the minute amounts of PIC proteins in an infected cell and the several cellular proteins that can bind non-specifically to DNA or biotin or streptavidin [10]. To identify the proteins associated specifically with PIC, the protein bands ranging in size from 10 kDa to 250 kDa were sliced into ~ 25 individual gel pieces and subjected to semi-quantitative MS analysis. The false discovery rate as determined using Peptide and Protein Prophet methods was less than 0.6% for all proteins identified. Given the reduced sample complexity, undersampling was not observed.

**Figure 3:**
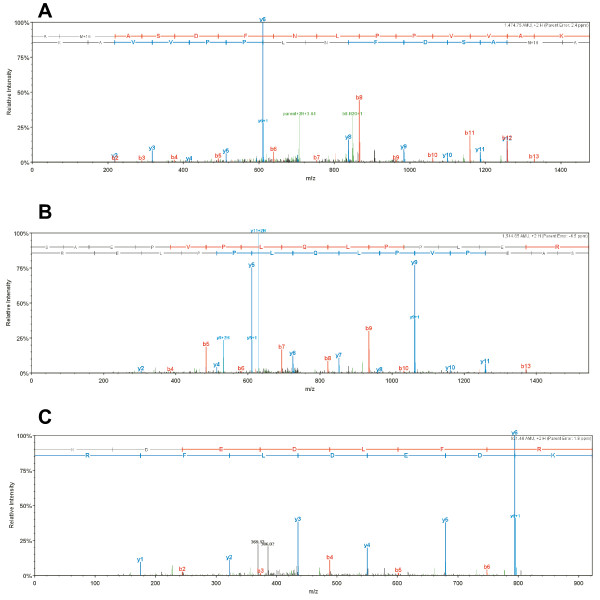
**Representative MS/MS data for HIV-1 and host proteins associated with PICs**. (A) HIV-1 integrase peptide AMASDFNLPPVVAK. (B) HIV-1 Rev peptide SAEPVPLQLPPLER. (C) Cellular barrier-to-autointegration factor (BAF) peptide KDEDLFR. The 'b" and "y" ion series derived from the amide bond cleavage during collision induced dissociation of the peptide provide amino acid sequence information. The b-ion series (shown in red) is read from the N-terminus to C-terminus, while the y-ion series (shown in blue) is read from the C-terminus to N-terminus, providing thus complementary sequence information [25]. Other minor fragments resulted from peptide fragmentations at other sites are shown in green.

**Figure 4:**
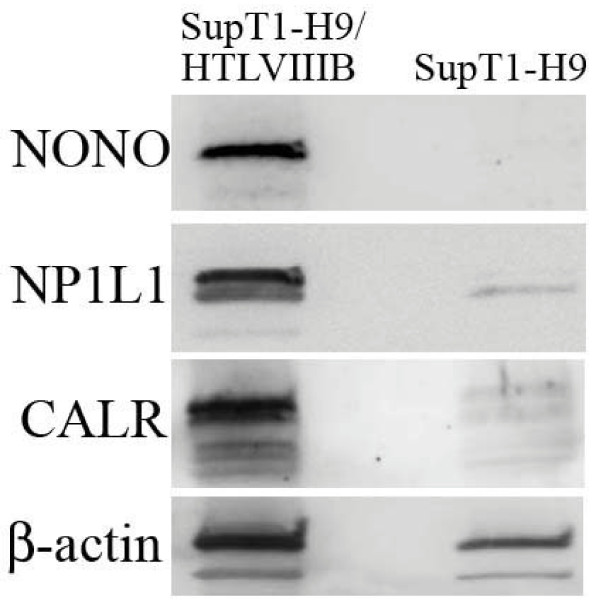
**Immunoblotting for host proteins that are specifically associated with HIV-1 PIC**. The proteins bound to the streptavidin magnetic beads after integration assay were probed with specific antibodies. SupT1-H9/HTLVIIIB represents HIV-1 infected cell samples, and SupT1-H9 represents non-infected control samples. The host proteins are indicated by accession names on the left. The 'NONO' is Non-POU domain-containing octamer-binding protein, 'NP1L1' is Nucleosome assembly protein 1-like 1 protein and 'CALR' is Calreticulin for which 8, 5 and 5 peptides were identified by MS analysis respectively. Beta-actin found in both samples is also shown.

The output from the MS analysis of two independent experiments of SupT1-H9/HTLVIIIB co-culture infections was compared against that of the SupT1-H9 co-culture control. The list of proteins present in SupT1-H9 co-culture experiment serves to eliminate the non-specifically binding cytoplasmic proteins from those identified in the SupT1-H9/HTLVIIIB co-culture samples. For a more sensitive and accurate analysis of the proteins associated with HIV-1 PICs, the following criteria have been employed: (a) identification of at least two peptides from each protein, and (b) identification of the protein in two independent SupT1-H9/HTLVIIIB co-culture samples. A total of 19 host proteins (~ 6% of the total proteins revealed by the MS analysis) were identified to be specifically associated with the HIV-1 PICs (Table 2). While barrier-to-autointegration factor (BAF) is the only host protein that was characterized previously [11,12], the identification of 18 new host proteins associated with HIV-1 PICs reflects the uniqueness of our approach. Two previously characterized proteins, Importin 7 [13] and Gemin2 [14] were detected in one of the two SupT1-H9/HTLVIIIB co-culture samples, cautioning that some of the characterized and uncharacterized proteins associated with PICs might not have been identified due to detection limits of MS. Lamina-associated polypeptide 2 isoform alpha (LAP2α) protein [15] was identified in the SupT1-H9/HTLVIIIB co-culture samples; however, its presence in SupT1-H9 samples suggests a non-specific interaction with biotinylated DNA. The integrase interacting protein, LEDGF/p75 (lens epithelium-derived growth factor), was not identified in the cytoplasmic PICs. The current analysis is limited to the identification of proteins associated with PIC assembling in the cytoplasm. A similar analysis of nuclear PICs is expected to reveal proteins such as LEDGF/p75 that function at the site of integration in the nucleus [16]. Importantly, the peptides corresponding to two HIV-1 proteins, IN and Rev were identified in SupT1-H9/HTLVIIIB co-culture samples (Figure 3). Recently, Rev has been suggested to regulate HIV-1 integration in infected cells based on its ability to interact with both IN and LEDGF/p75 [17]. Rev could therefore potentially contribute to the formation of PICs. Figure 4 shows a representative immunoblotting of SupT1-H9 and SupT1-H9/HTLVIIIB samples confirming the host proteins that specifically associated with HIV-1 PICs.

**Table 2:** Host proteins selectively co-purifying with HIV-1 PICs

*Host Proteins*	*Accession numbers*	*Molecular Weight*	*No. of peptides*
***Chromatin organization***			

Barrier-to-autointegration factor	baf_bovin	10 kDa	3

Nucleosome assembly protein 1-like 1	np1l1_bovin	45 kDa	5

Histone-binding protein RBBP4	rbbp4_bovin	48 kDa	3

***Transcription regulation ***			

Acidic leucine-rich nuclear phosphoprotein 32 family member A	an32a_bovin	29 kDa	4

Acidic leucine-rich nuclear phosphoprotein 32 family member E	an32e_human	31 kDa	3

Calreticulin	calr_cerae	48 kDa	5

NF-kappa-B essential modulator	nemo_bovin	49 kDa	7

Non-POU domain-containing octamer-binding protein	nono_human	54 kDa	8

RNA polymerase-associated protein LEO1	leo1_human	75 kDa	6

***RNA processing/localization***			

ATP-dependent RNA helicase DDX19A	dd19a_bovin	54 kDa	4

Double-stranded RNA-binding protein Staufen homolog 1	stau1_human	63 kDa	2

Heterogeneous nuclear ribonucleoprotein H1	hnrh1_human	49 kDa	2

Heterogeneous nuclear ribonucleoprotein H3	hnrh3_human	37 kDa	3

Plasminogen activator inhibitor 1 RNA-binding protein	pairb_human	45 kDa	7

Splicing factor 3B subunit 2	sf3b2_human	98 kDa	19

Splicing factor, arginine/serine-rich 3	sfrs3_bovin	19 kDa	3

U4/U6.U5 tri-snRNP-associated protein 1	snut1_human	90 kDa	17

***Translation***			

Eukaryotic translation initiation factor 4 gamma 1	if4g1_human	176 kDa	3

***Cytoplasmic trafficking***			

Dynactin subunit 2	dctn2_human	44 kDa	3

The host proteins have been broadly categorized based on the known physiological function. The number of peptides identified by mass spectroscopic analysis and assigned to the specific protein in the database is shown.

Of the host proteins identified here to be specifically associated with HIV-1 PICs, histone-binding protein RBBP4 is known to influences transcription activation by facilitating histone acetylation [18], and non-POU domain-containing octamer-binding protein is characterized to function with respect to double strand DNA break repair [19]. Nucleosome assembly protein 1-like 1 protein has been shown to interact with HIV-1 Tat and promote viral transcription [20], while splicing factor 3B subunit 2 protein interacts with HIV-1 Vpr and activates G2 checkpoint activation [21]. Moreover, the double-stranded RNA-binding protein Staufen homolog 1 is incorporated in HIV-1 and plays a role in viral genomic RNA encapsidation and viral particle assembly [22-24]. It is tempting to speculate that such factors might play a role in the assembly of PICs and assist the formation of proviral DNA similar to that of BAF or LEDGF/p75 and fulfill the roles not attributed to the previously characterized host factors. A detailed characterization of the host proteins identified here is essential to elucidate their role in HIV integration and to verify their potential utility as cellular targets for drug development. In conclusion, the approach described here for the identification of host proteins associated with HIV-1 PIC revealed a number of previously not described host proteins which potentially contribute to HIV-1 integration. In addition, the application of the method depicted here could be used for characterizing nucleoprotein complexes from other retroviruses.

## List of abbreviations

IN: integrase; PIC: preintegration complex; HAT: histone acetyltransferase; HMGA1: high mobility group A1; HSP 60: heat shock protein 60; EED: embryonic ectoderm development; LEDGF/p75: lens epithelium-derived growth factor; UNG2: uracil-DNA glycosylase 2.

## Competing interests

The authors declare that they have no competing interests.

## Authors' contributions

NKR conceived the study, designed and performed the biochemical experiments and drafted the manuscript. SH and MK designed, and NS and RLJG performed the mass spectrometry and helped in drafting the manuscript. LW coordinated the study, participated in the experimental design and the drafting of the manuscript. All authors read and approved the final manuscript.
